# Supplementary material for: A Critical View of the Application of the APEX Software (Aqueous Photochemistry of Environmentally-Occurring Xenobiotics) to Predict Photoreaction Kinetics in Surface Freshwaters
Source: Molecules. 2019 Dec 18;25(1):9. doi: 10.3390/molecules25010009 (PMC7017383; doi:10.3390/molecules25010009)
Supplement: Supplementary file 1 [file molecules-25-00009-s001.zip › 3.2.4_gcc-4.4.0.zip!/3.2.4_gcc-4.4.0/3.2.4_gcc-4.4.0/doc/octave/html/interpreter/Output-Conversion-Syntax.html]

Output Conversion Syntax - Untitled


Next: Table of Output Conversions,
Previous: Output Conversion for Matrices,
Up: C-Style I/O Functions


---

#### 14.2.6 Output Conversion Syntax

This section provides details about the precise syntax of conversion
specifications that can appear in a `printf` template
string.

Characters in the template string that are not part of a
conversion specification are printed as-is to the output stream.

The conversion specifications in a `printf` template string have
the general form:

```
     % flags width [ . precision ] type conversion
```

For example, in the conversion specifier %-10.8ld, the -
is a flag, 10 specifies the field width, the precision is
8, the letter l is a type modifier, and d specifies
the conversion style. (This particular type specifier says to print a
numeric argument in decimal notation, with a minimum of 8 digits
left-justified in a field at least 10 characters wide.)

In more detail, output conversion specifications consist of an
initial % character followed in sequence by:

- Zero or more flag characters that modify the normal behavior of
  the conversion specification.
  - An optional decimal integer specifying the minimum field width.
    If the normal conversion produces fewer characters than this, the field
    is padded with spaces to the specified width. This is a *minimum*
    value; if the normal conversion produces more characters than this, the
    field is *not* truncated. Normally, the output is right-justified
    within the field.
    You can also specify a field width of \*. This means that the
    next argument in the argument list (before the actual value to be
    printed) is used as the field width. The value is rounded to the
    nearest integer. If the value is negative, this means to set the
    - flag (see below) and to use the absolute value as the field
    width.- An optional precision to specify the number of digits to be
      written for the numeric conversions. If the precision is specified, it
      consists of a period (.) followed optionally by a decimal integer
      (which defaults to zero if omitted).
      You can also specify a precision of \*. This means that the next
      argument in the argument list (before the actual value to be printed) is
      used as the precision. The value must be an integer, and is ignored
      if it is negative.- An optional type modifier character. This character is ignored by
        Octave's `printf` function, but is recognized to provide
        compatibility with the C language `printf`.- A character that specifies the conversion to be applied.

The exact options that are permitted and how they are interpreted vary
between the different conversion specifiers. See the descriptions of the
individual conversions for information about the particular options that
they use.
